# MdJa2 Participates in the Brassinosteroid Signaling Pathway to Regulate the Synthesis of Anthocyanin and Proanthocyanidin in Red-Fleshed Apple

**DOI:** 10.3389/fpls.2022.830349

**Published:** 2022-05-09

**Authors:** Mengyu Su, Shuo Wang, Wenjun Liu, Ming Yang, Zongying Zhang, Nan Wang, Xuesen Chen

**Affiliations:** ^1^State Key Laboratory of Crop Biology, College of Horticulture Science and Engineering, Shandong Agricultural University, Tai’an, China; ^2^Collaborative Innovation Center of Fruit & Vegetable Quality and Efficient Production in Shandong, Tai’an, China; ^3^College of Continuing Education, Shandong Agricultural University, Tai’an, China

**Keywords:** red-fleshed apple, brassinosteroid, MdJa2, MdBZR1, anthocyanin, proanthocyanidin

## Abstract

Anthocyanin and proanthocyanidin play important roles in plant secondary metabolism. Although previous studies identified many transcription factors involved in anthocyanin and proanthocyanidin synthesis, the effects of MADS-box transcription factors are unclear in apple. Brassinosteroids (BRs) are steroid hormones that affect plant flavonoid biosynthesis, but the underlying regulatory mechanism is not yet well established. In this study, we identified a MADS-box transcription factor, MdJa2, which contained a highly conserved MADS-box domain and belonged to the STMADS11 subfamily. Additionally, *MdJa2* was responsive to BR signal, and the overexpression of *MdJa2* inhibited the synthesis of anthocyanin and proanthocyanidin. The silencing of *MdJa2* in “Orin” calli promoted anthocyanin and proanthocyanidin accumulations. Moreover, MdJa2 interacted with MdBZR1. MdJa2 was revealed to independently regulate anthocyanin and proanthocyanidin synthesis pathways. The MdJa2–MdBZR1 complex enhanced the binding of MdJa2 to the promoters of downstream target genes. Our research provides new insights into how MADS-box transcription factors in the BR signaling pathway control the accumulations of anthocyanin and proanthocyanidin in red-fleshed apple.

## Introduction

Apple, which is one of the main fruit cash crops in temperate regions, is an important part of the fruit tree industry in China. The twigs and young leaves of red-fleshed apple trees are red-to-purplish red, whereas their flowers are bright purplish red and their fruits remain purplish red as they develop. These trees are relatively rare and considered attractive because of their leaf, flower, and fruit colors. The coloration degree of the red-fleshed apple fruit is closely related to the anthocyanin contents. Anthocyanins are a kind of flavonoids, which are important for secondary metabolism as well as growth and development. Flavonoids have been divided into the following three main categories: flavonols, anthocyanins, and proanthocyanidins (PAs; Williams and Grayer, 2004). Like anthocyanins, PAs and flavonols also have important functions (Dixon, 1986; Harborne and Williams, 2000).

Previous studies revealed that the synthesis and accumulations of anthocyanin and PA in plants required multiple related enzymes and transcription factors. The genes involved in anthocyanin and PA biosynthesis pathways in apple can be divided into the following two categories: structural genes and regulatory genes. Structural genes are those that encode enzymes that catalyze reactions in flavonoid synthesis pathways, including chalcone synthase (*CHS*), chalcone isomerase (*CHI*), flavanone 3-hydroxylase (*F3H*), dihydroflavonol 4-reductase (*DFR*), anthocyanidin synthase (*ANS*), flavonoid 3-O-glycosyltransferase (*UFGT*), leucoanthocyanidin reductase (*LAR*), and anthocyanidin reductase (*ANR*; Koes et al., 2005; Hichri et al., 2011). The regulatory genes encoding transcription factors can modulate the expressions of structural genes. The transcription factors controlling these pathways are mainly from the MYB, bHLH, and WD40 families (Baudry et al., 2004; Quattrocchio et al., 2006; An et al., 2012; Xie et al., 2012). These transcription factors usually form MYB–bHLH–WD40 (MBW) complex that directly regulate the transcriptions of structural genes. In apple, MdMYBA/1/10, MdMYB3, MdbHLH3, MdbHLH33, and MdTTG1 form a MYB–bHLH–WD40/WDR complex, which is involved in the regulation of anthocyanin and PA levels (Takos et al., 2006; Espley et al., 2007; Lin-Wang et al., 2011; Xie et al., 2012; Bai et al., 2014). Moreover, MdMYB9 and MdMYB11 can bind directly to the *LAR*, *ANR*, and *ANS* promoters, whereas MdbHLH3 is recruited upstream of *MdMYB9* and *MdMYB11* to regulate the transcriptions of the corresponding genes (An et al., 2015).

In addition to the MBW complex, other transcription factors also affect anthocyanin and PA biosynthesis. For example, MdNAC52 can regulate the biosynthesis of anthocyanin and PA by interacting with the promoters of *MdMYB9* and *MdMYB11* (Sun et al., 2019). The ethylene response factor MdERF1B can regulate anthocyanin and PA biosynthesis in apple by directly binding to the promoters of *MdMYB9* and *MdMYB11* (Zhang et al., 2018). Additionally, MdEIL1 functions upstream of *MdMYB1* and can activate its expression, leading to anthocyanin biosynthesis (An et al., 2018). HYPOCOTYL5 (HY5) is a positive regulator of light-induced photomorphogenesis and anthocyanin biosynthesis (Gangappa and Botto, 2016). The overexpression of the *Brassica napus* gene *BnWRKY41* in *Arabidopsis* decreases the anthocyanin contents (Duan et al., 2018). In response to ultraviolet radiation and low-temperature stress, *MdBBX20* promotes anthocyanin accumulations (Fang et al., 2019). The overexpression of the kiwifruit *SVP3* gene suppresses anthocyanin biosynthesis in petals (Wu et al., 2014). Jaakola et al. (2010) reported that a *SQUAMOSA* MADS-box gene regulated anthocyanin accumulations in bilberry (*Vaccinium*) fruit (Jaakola et al., 2010). Wu et al. (2013b) concluded that a MADS-box transcription factor likely contributed to anthocyanin biosynthesis in the European pear (Wu et al., 2013b). The STMADS subfamily genes have been reported to function as growth inhibitors that help to maintain dormancy in perennial woody plants (Mazzitelli et al., 2007; Bielenberg et al., 2008; Diaz-Riquelme et al., 2009; Horvath et al., 2010; Li et al., 2010; Yamane et al., 2011). The deletion of the six *SVP*-like *DORMANCY-ASSOCIATED MADS-BOX* (*DAM*) genes in peach (*Prunus persica*) results in the failure to enter dormancy following an exposure to cold or short-day conditions (Bielenberg et al., 2008; Yamane et al., 2011). In apple, the STMADS subfamily genes primarily influence plant growth and development, but their effects on anthocyanin and PA synthesis have rarely been studied.

In addition to being affected by the genotype, anthocyanin and PA biosynthesis are also modulated by external factors, including light and temperature, as well as by hormone and sugar contents (Winkel-shirley, 2001; Lepiniec et al., 2006; Takos et al., 2006). Most of the external stimuli regulate the synthesis of anthocyanin and PA by altering the expression of structural genes or related transcription factor genes. Brassinolide (BL) is the most active brassinosteroid (BR), which is one of the six main types of plant hormones (Grove et al., 1979). Brassinosteroids control plant photomorphogenesis, cell elongation and division, leaf and plant morphogenesis, pollen fertility, and plant yield and quality (Li et al., 1996a; Li and Chory, 1997; Clouse, 2011). Recently, BR has been reported to be involved in flavonoid metabolism in *Arabidopsis* and apples (Liang et al., 2020; Wang et al., 2021). However, whether STMADS subfamily genes in red-fleshed apples participate in the BR signaling pathway to regulate anthocyanin and PA synthesis remains unknown.

In this study, we used molecular and genetic methods to study the STMADS11 subfamily gene MdJa2 regarding its involvement in the BR pathway and its regulatory effects on the synthesis of anthocyanin and PA in red-fleshed apple. We observed that MdJa2 mainly repressed anthocyanin and PA accumulations by binding to the *MdANS*, *MdMYB9,* and *MdMYB12* promoters. Brassinosteroids could induce the production of *MdJa2*. The transcription factor MdBZR1, which is an important part of the BR signaling pathway, and MdJa2 could form a complex that regulated the synthesis of anthocyanin and PA. The objectives of this study were to clarify how MADS-box transcription factors affect the BR signaling pathway to regulate anthocyanin and PA biosynthesis, which is currently unknown.

## Materials and Methods

### Plant Materials

The red-fleshed apple calli (Wang et al., 2021) were cultured on Murashige and Skoog (MS) medium containing 1 mg L^−1^ 6-benzylaminopurine (6-BA) and 0.3 mg L^−1^ 1-naphthylacetic acid. The red-fleshed apple seedlings (Wang et al., 2021) were grown on MS medium containing 0.2 mg L^−1^ indoleacetic acid and 0.5 mg L^−1^ 6-BA. The calli and seedlings were cultured under long-day conditions (16-h light/8-h dark; 50 μmol m^−2^ s^−1^ light intensity) at 24°C. The calli and seedlings were subcultured every 15 and 30 days, respectively. “Orin” calli were grown on MS medium supplemented with 0.5 mg L^−1^ 6-BA and 1 mg L^−1^ 2,4-dichlorophenoxyacetic acid at 24°C in darkness. Additionally, they were subcultured every 15 days. Regarding the hormone experiment, the red-fleshed apple seedlings were grown for 7 days on medium supplemented with 1 μM BL. The red-fleshed apple calli were grown for 14 days on medium supplemented with 1 μM BL.

### Measurement of the Anthocyanin Content

Each sample (1 g) was ground to powder in liquid nitrogen, after which 0.5 g ground material was added to 20 ml 1% HCl–methanol and incubated at 4°C for 24 h. The sample was then centrifuged at 12,000 rpm for 15 min. The optical density of the supernatant was measured at 510 and 700 nm (i.e., OD_510_ and OD_700_, respectively) using the UV-2450 spectrophotometer (Shimadzu, Kyoto, Japan). The anthocyanin content was calculated using the following equation: ΔA × 5 × 0.005 × 1,000 × 449.2/(26,900 × 0.5), where Δ*A* = (OD_510_ − OD_700_ at pH 1.0) − (OD_510_ − OD_700_ at pH 4.5).

### Total RNA Extraction and Quantitative Real-Time Polymerase Chain Reaction

Total RNA was extracted using the RNAprep Pure Plant kit (Tiangen, Beijing, China) and then reverse transcribed to cDNA using the RevertAid^™^ First Strand cDNA Synthesis Kit (TransGen, Beijing, China). A quantitative real-time polymerase chain reaction (qRT-PCR) analysis was performed using the SYBR^®^ Green PCR Master Mix (TransGen) and the CFX96 system (Bio-Rad, Hercules, CA, United States). Three replicates were analyzed per sample. The *MdActin* gene was used as an internal control. Relative mRNA levels were calculated according to the 2^−ΔΔCt^ method (Livak and Schmittgen, 2001). The qRT-PCR primers are listed in Supplementary Table S2.

### Transformation of Apple Calli

For the gene transformation experiment, *MdJa2* (LC004729.1) and *MdBZR1* (LOC103440434) were inserted into the pRI101-AN vectors containing the GFP tag and the CaMV 35S promoter. Additionally, *MdJa2*_271-672bp_ was inserted into the pFGC1008 vector for the gene silencing analysis. Well-grown calli were infected with *Agrobacterium tumefaciens* LBA4404 for 30 min at room temperature, and the infected calli were co-cultured on MS solid medium at 24°C for 1–2 days in the dark. The calli infected with the *MdJa2-PRI* and *MdBZR1-PRI* recombinant plasmids were transferred to the selection medium containing 50 mg L^−1^ kanamycin and 250 mg L^−1^ carbenicillin, and the calli infected with the MdJa2-RNAi recombinant plasmid were transferred to the selection medium containing 250 mg L^−1^ carbenicillin and 100 mg L^−1^ hygromycin-B. The presence of the transgene was confirmed by PCR amplification, western blotting and qRT-PCR. Regarding the hormone experiment, the apple calli were grown for 14 days on the medium supplemented with 1 μM BL.

### Western Blotting and Related Antibodies

Western blotting was performed using a 0.45 μm nitrocellulose membrane. The components and amounts of the transfer solution were as follows: Tris-base 3 g, glycine 14.4 g, methanol 200 ml, and ddH_2_O 800 ml. The current was set to 120 mA, and electrophoresis was carried out in an ice-water bath for 3 h. After electrophoresis, the membrane was shaken and blocked in PBST buffer (2 g skimmed milk powder + 40 ml phosphate-buffered saline with Tween 20 buffer) for 1 h at 4°C. The blocking solution was discarded, the primary antibody diluent (6 μl primary antibody diluted in 30 ml blocking solution) was poured into the nitrocellulose membrane, and the nitrocellulose membrane was incubated at 4°C for 10–12 h with shaking. After the primary antibody incubation, the secondary antibody diluent (4 μl secondary antibody diluted in 30 ml blocking solution) was poured into the nitrocellulose membrane, the nitrocellulose membrane was incubated with shaking at 4°C for 3 h. Then the nitrocellulose membrane was rinsed with developing solution, and developed on a Chemi DOC chemiluminescence imager (BIO-RAD). GFP-anti (GFP: green fluorescent protein, anti: antibody) primary antibody was used for detection of transgenic calli, HIS (His: histidine)-anti and GST (GST: glutathione S-transferase)-anti primary antibodies were used in the following pull-down assays, and goat anti-mouse secondary antibodies were used in the detection of transgenic calli and pull-down assays.

### Measurement of the PA Content by Staining With 4-Dimethylaminocinnamaldehyde

The presence of PA was detected on the basis of 4-Dimethylaminocinnamaldehyde (DMACA) staining. Specifically, calli were stained with the DMACA reagent (0.2% DMACA, w/v, methanol: 6 M HCl, *v/v* = 1:1) for 30 min and then washed with 70% (*v/v*) ethanol. The PA content was measured as described by Li et al. (1996b). Each sample was ground to powder in liquid nitrogen. Next, 0.4 g powder was resuspended in 1 ml 70% (*v/v*) acetone solution containing 0.1% (*v/v*) ascorbic acid and mixed for 30 min at 4°C in darkness. These steps were repeated three times and the supernatant was collected by centrifugation. The supernatant (4 ml) was mixed with 3 ml ether at −20°C. Soluble PAs were contained in the lower layer of the resulting solution. A 770 μl aliquot of the soluble PAs was mixed with 385 μl methanol and 192 μl 2% (w/v) DMACA at room temperature for 20 min, after which the absorbance at 643 nm was measured using the UV-2450 spectrophotometer (Shimadzu, Kyoto, Japan). The PA concentration was calculated according to a standard curve prepared using catechin.

### Yeast Two-Hybrid Assays

The MdJa2 cDNA sequences (full-length, N-terminus, and C-terminus) were inserted into the pGBKT7 vectors. Additionally, the MdBZR1 CDS was incorporated into the pGADT7 vector. The yeast two-hybrid (Y2H) assays were performed according to the manufacturer’s instructions (Clontech, United States). To detect self-activation, the Y2H Gold yeast strains containing the recombinant pGBKT7 vectors were grown on medium lacking Trp and Leu (−T/−L). To screen for protein–protein interactions, yeast strains containing the recombinant pGADT7 and pGBKT7 vectors were grown on medium lacking Trp, Leu, His, and Ade (−T/−L/−H/−A) with or without X-α-gal. The empty pGADT7 and pGBKT7 vectors were used as controls. The primers used are listed in Supplementary Table S1.

### Pull-Down Assays

The full-length of MdJa2 was inserted into the pET32a (+) vector, whereas the MdBZR1 CDS was inserted into the pGEX-4 T-1 vector (Novagen).^1^ The two recombinant plasmids were used to transform *Escherichia coli* BL21 (DE3) for the expression of MdJa2-histidine (His) and MdBZR1-glutathione S-transferase (GST) fusion proteins. Next, MdJa2-His was incubated with MdBZR1-GST or GST alone. After an immunoprecipitation with an anti-His column, the eluted protein mixture was analyzed by immunoblotting using an anti-GST antibody (Clontech).

### Bimolecular Fluorescence Complementation Assays

The *MdJa2* CDS was inserted into the pSPYNE-35S vector, whereas the *MdBZR1* CDS was inserted into the pSPYCE-35S vector. *A. tumefaciens* LBA4404 cells were transformed with the recombinant plasmids and then cultured in YEP medium until the optical density at 600 nm (OD_600_) reached 0.6. The pSPYNE-35S and pSPYCE-35S bacterial solutions were mixed (15 ml each) and used to infect onion epidermal cells, which were then cultured in darkness at 28°C for 24–48 h. Finally, YFP fluorescence was observed using the BX53F confocal laser scanning microscope (Olympus, Tokyo, Japan).

### Yeast One-Hybrid Assays

MdJa2 was inserted into the pGADT7 vector. The *MdANS*, *MdMYB9* and *MdMYB12* promoter fragments were inserted into the pHIS2 vectors (BD Biosciences, Shanghai, China). The background histidine leakage from the pHIS2 vector was inhibited using 3-amino-1,2,4-triazole (3-AT). To select the optimal 3-AT concentration, Y187 (Clontech) yeast strains containing the *MdANS-pHIS2*, *MdMYB9-pHIS2,* and *MdMYB12-pHIS2* recombinant plasmids were grown on medium lacking Trp and His (−T/−H). The protein–DNA interactions were then tested on selective medium lacking Trp, Leu, and His (−T/−L/−H), but supplemented with the optimal 3-AT concentration. An empty pGADT7 vector was used as a control.

### Electrophoretic Mobility Shift Assays (EMSAs)

MdJa2 was incorporated into the pET32a vector (Novagen),^2^ which includes a His tag sequence. The recombinant plasmid was inserted into *E. coli* BL21 (TransGen) cell for the production of the fusion protein, which was then purified using the His-tagged Protein Purification kit (CWBIO Inc., Beijing, China). All probes for the promoter fragments were labeled and synthesized by the Sangon Biotechnology Co., Ltd. (Supplementary Table S3). The EMSA was performed using the LightShift Chemiluminescent EMSA Kit (Thermo Scientific, Waltham, MA, United States). The reaction mixture contained 2 μl 1× binding buffer (2.5% glycerol, 5 mM MgCl_2_, 10 mM EDTA, and 50 mM KCl), 17 μl protein, and 1 μl probe. The mixture was incubated at room temperature for 30 min.

### Luciferase (LUC) Reporter Assays

The *MdJa2* and *MdBZR1* CDSs were inserted into the pHBT-AvrRpm1-HA vector, pHBT-AvrRpm1-Flag vector, respectively (i.e., effectors), whereas the *MdANS*, *MdMYB9,* and *MdMYB12* promoters were inserted into the pFRK1-LUC-NOS vectors (i.e., reporters). Protoplasts of “Orin” calli were extracted with enzymatic hydrolysis solution (containing pectinase and cellulase). Regarding the transient expression, apple protoplasts were transformed with a solution containing 12 μg effector plasmid, 6 μg luciferase (LUC) reporter plasmid, and 2 μg GUS plasmid. The transiently transfected protoplasts were incubated at 24°C for 6 h. The samples were added 100 μl cell lysate and vortexed vigorously for 10 s, which were placed on ice. A 96-well white plate was used, each well was added 100 μl luciferase test substrate, then added 20 μl of the above cell extract, gently shaked and mixed, and the LUC value was immediately readed according to the set program. A 96-well black plate was using, each well was added 20 μl MUG buffer, then added 5 μl of the above cell extract, gently shaked and mixed, and incubated at 37°C for 1 h in the dark. Then the mix well was added 100 μl 0.2M Na_2_CO_3_ to stop the reaction, and the GUS value was readed according to the set program. The GUS and LUC activities were detected using the VictorX4 Multimode Plate Reader (PerkinElmer, Waltham, MA, United States). The promoter activity was quantified according to the LUC/GUS activity ratio.

### Data Analysis

All datas are analyzed with Tukey’s test. These values are the mean ± SDs of three independent biological replicates.

## Results

### BR Treatment Inhibits the Synthesis of Anthocyanin and PA in Red-Fleshed Apples

**Figure 1:**
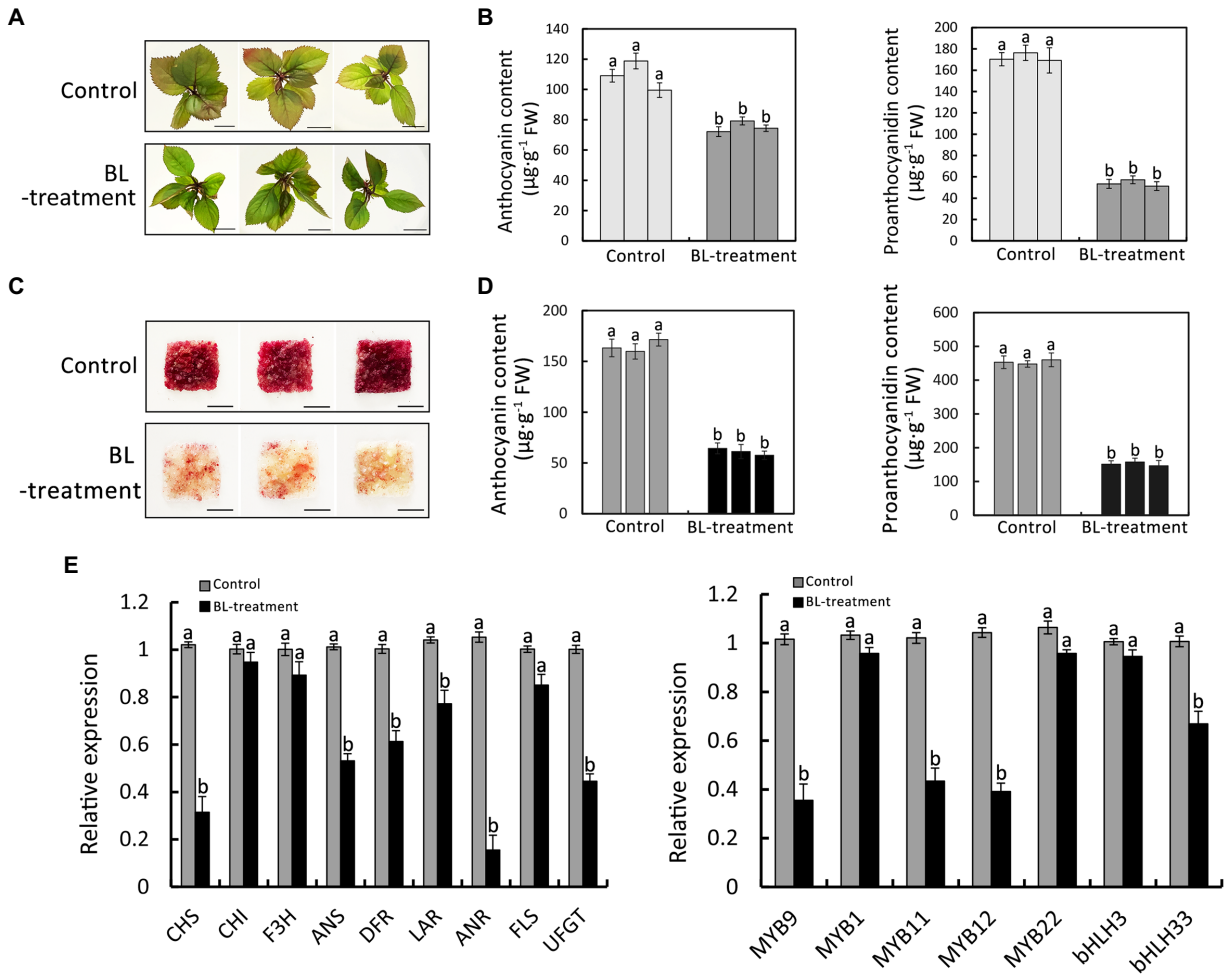
Effect of BR on the biosynthesis of anthocyanin and proanthocyanidin in red-fleshed apple seedlings and calli. **(A)** Phenotype of red-fleshed apple seedlings treated with 1 μM BL for 7 days. Scale bar = 1 cm. **(B)** Anthocyanin and proanthocyanidin contents in red-fleshed apple seedlings. **(C)** Phenotype of red-fleshed apple calli treated with 1 μM BL for 14 days. Scale bar = 1 cm. **(D)** Anthocyanin and proanthocyanidin contents in red-fleshed apple calli. **(E)** Results of the qRT-PCR analysis of the expressions of flavonoid synthesis-related genes in red-fleshed apple seedlings after the BL treatment. Statistical significance is indicated by different lowercase letters (*p < 0.05*).

To ascertain the effect of BR on anthocyanin and PA synthesis, red-fleshed apple seedlings and calli were treated with 1 μmol BL (BR signal activator) according to previous research (Wang et al., 2021). After a 7- and 14-day of culture under normal conditions, the BL-treated red-fleshed apple seedlings and calli were less red than the control (Figure 1A,C). The subsequent analysis of the anthocyanin and PA contents of the red-fleshed apple seedlings and calli revealed that BL inhibited the accumulations of anthocyanin and PA (Figure 1B,D). We also examined the expression levels of flavonoid synthesis genes and the related transcription factor genes in red-fleshed apple seedlings treated with BL. The BL treatment significantly inhibited the expression of the flavonoid synthesis genes (*MdANS*, *MdDFR*, *MdANR*, and *MdUFGT*) and the related transcription factor genes (*MdMYB9*, *MdMYB11*, and *MdMYB12*; Figure 1E). Therefore, the BR signal might be involved in the synthesis of anthocyanin and PA in red-fleshed apple.

### *MdJa2* Is Responsive to the BR Signal and *MdJa2* Overexpression Inhibits Anthocyanin and PA Synthesis

**Figure 2:**
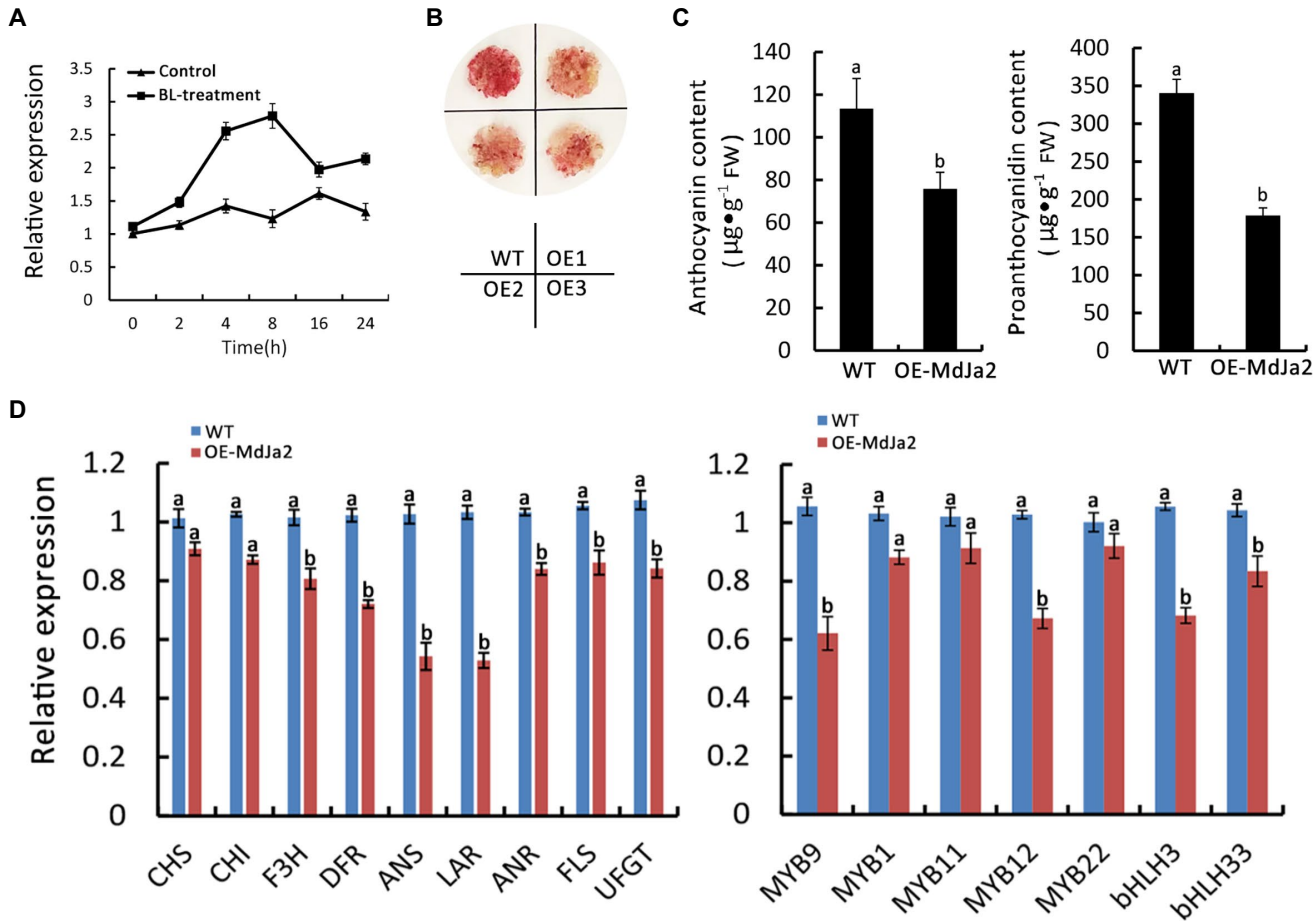
*MdJa2* responds to the BR signal and negatively regulates the synthesis of anthocyanin and proanthocyanidin in red-fleshed apple calli. **(A)** The expression of *MdJa2* in red-fleshed apple seedlings treated with BL as determined by qRT-PCR. **(B)** Phenotypes of *OE-MdJa2* red-fleshed apple calli. **(C)** Anthocyanin and proanthocyanidin contents in wild-type (WT) and transgenic lines. **(D)** Expressions of the genes related to flavonoid synthesis in *OE-MdJa2* red-fleshed apple calli as determined by qRT-PCR. Statistical significance is indicated by different lowercase letters (*p < 0.05*).

The phylogenetic analysis of MdJa2 and selected MADS-box family genes from *Arabidopsis* and rice indicated that the red-fleshed apple *MdJa2* gene belonged to the STMADS11 subfamily (Supplementary Figure S1). To determine whether *MdJa2* is involved in the BR signaling pathway, the *MdJa2* expression level in BL-treated red-fleshed apple seedlings was analyzed. The data indicated that *MdJa2* expression was induced by BR (Figure 2A). To functionally characterize *MdJa2* regarding its role in the synthesis of anthocyanin and PA in red-fleshed apple, we introduced the *MdJa2* overexpression vector (*OE-MdJa2*) into wild-type (WT) red-fleshed apple calli. Three independent *MdJa2*-overexpressing calli were selected for the gene expression analysis (Supplementary Figure S2). Compared with the WT red-fleshed apple calli, the *MdJa2*-overexpressing calli were lighter red (Figure 2B). Moreover, the overexpression of *MdJa2* resulted in decreased anthocyanin and proanthocyanidin contents (Figure 2C). To clarify the transcriptional regulatory effect of *MdJa2* on genes in the flavonoid synthesis pathway, we analyzed the expression levels of flavonoid synthesis-related genes and transcription factor genes in *OE-MdJa2* red-fleshed apple calli. Compared with the WT, the expression levels of flavonoid synthesis structural genes (*MdANS* and *MdLAR*) and transcription factors (*MdMYB9*, *MdMYB12,* and *MdbHLH3*) were down-regulated in the *OE-MdJa2* transgenic calli (Figure 2D).

To further elucidate the role of *MdJa2* in the anthocyanin and proanthocyanidin synthesis pathways, we introduced the *MdJa2*-RNAi recombinant plasmid into the WT “Orin” calli to silence the expression of *MdJa2*. Three independent *MdJa2*-silenced calli were examined regarding their phenotypes. Compared with the WT calli, the *MdJa2-RNAi* calli were more intensely red (Supplementary Figure S3A) under light and contained higher anthocyanin and proanthocyanidin contents (Supplementary Figure S3B). We subsequently determined the expression levels of flavonoid synthesis-related genes and transcription factor genes in the *MdJa2*-RNAi calli. The flavonoid synthesis structural genes (*MdANS* and *MdDFR*) and transcription factor genes (*MdMYB9* and *MdMYB12*) were more highly expressed in the *MdJa2*-RNAi calli than in the WT calli (Supplementary Figure S3C). Therefore, the silencing of *MdJa2 via* RNAi in ‘Orin’ calli indicated this gene contributed to the synthesis of anthocyanin and proanthocyanidin.

### BR Inhibits the Synthesis of Anthocyanin and Proanthocyanidin in the Overexpression of *MdJa2* Red-Fleshed Apple Calli

**Figure 3:**
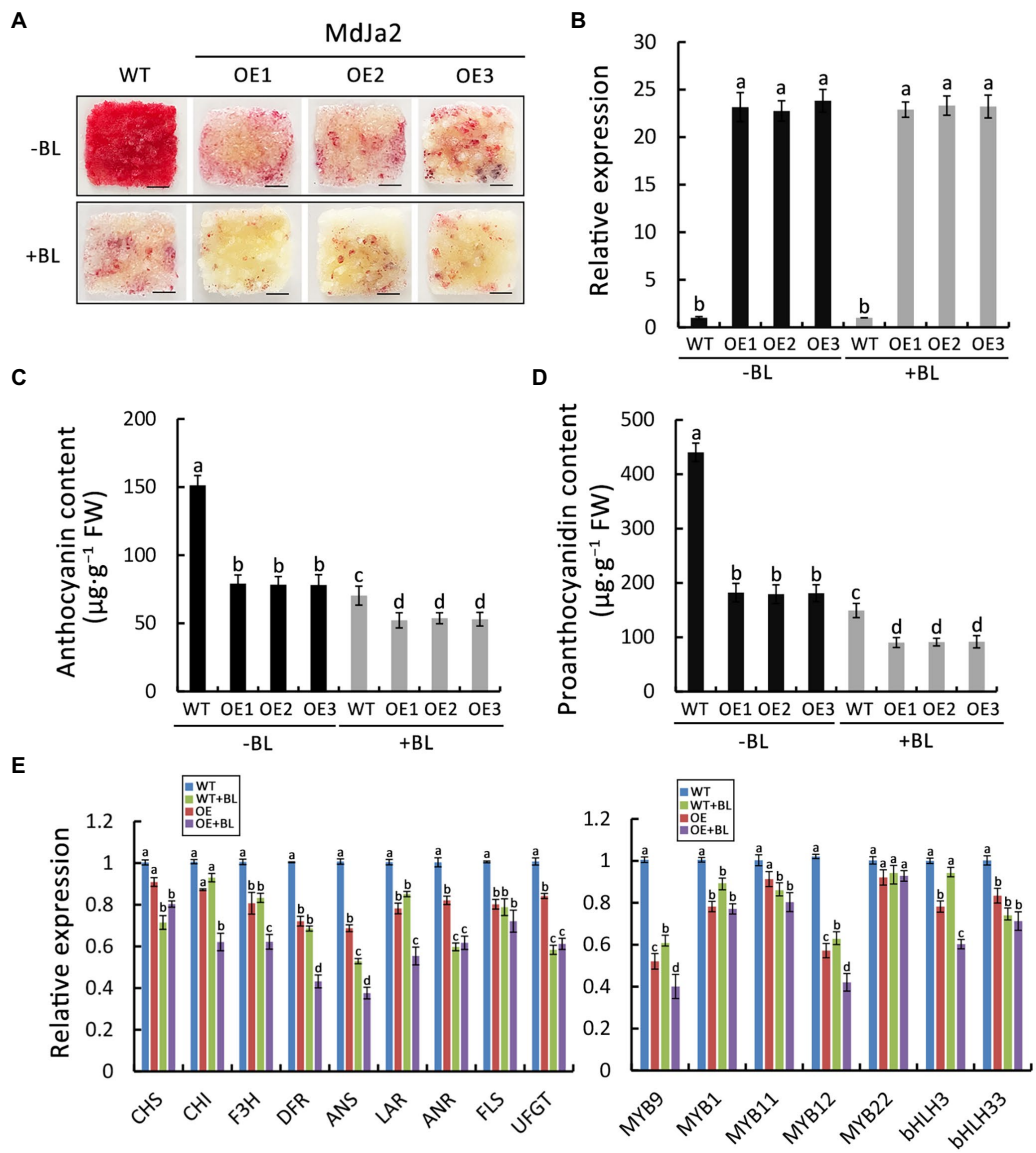
Effect of BL-treatment on anthocyanin and proanthocyanidin synthesis in *OE-MdJa2* red-fleshed apple calli. **(A)** Phenotypes of *MdJa2*-overexpressing (*OE-MdJa2*) apple calli grown on MS medium with or without 1 μM BL. The experiment was performed at least three times. Scale bar = 1 cm. **(B)** qPCR analysis of *MdJa2* transcript in WT and transgenic apple calli. **(C,D)** Anthocyanin and proanthocyanidin contents in wild-type (WT) and transgenic lines with or without BL-treatment. **(E)** Expressions of the genes related to flavonoid synthesis in *OE-MdJa2* and WT red-fleshed apple calli with or without 1 μM BL as determined by qRT-PCR. Statistical significance is indicated by different lowercase letters (*p < 0.05*).

To elucidate the effect of BR on the synthesis of anthocyanin and proanthocyanidin in *OE-MdJa2* red-fleshed apple calli, we used BL to treat the transgenic calli. Compared with the control, the redness of the calli overexpressing *MdJa2* had faded (Figure 3A). The presence of the transgene in *OE-MdJa2* apple calli was confirmed by qPCR analysis (Figure 3B). Moreover, the anthocyanin and proanthocyanidin contents decreased in the transgenic calli, especially in BL-treated transgenic calli (Figures 3C,D). Compared with WT control, *OE-MdJa2* transgenic calli after BL treatment, the expression levels of flavonoid synthetic structural genes (*MdDFR* and *MdANS*) and transcription factors (*MdMYB9* and *MdMYB12*) were down-regulated (Figure 3E). Next, we treated *MdJa2-RNAi* calli with BL, and *MdJa2-RNAi* calli were less pigmented after treatment with BL under continuous light compared with the control (Supplementary Figure S4A). The contents of the anthocyanin and proanthocyanidin were also reduced (Supplementary Figure S4B). We subsequently determined the expression levels of flavonoid synthesis-related genes in *MdJa2-RNAi* calli after treatment with BL. It was found that the flavonoid synthesis-related genes were further down-regulated in *MdJa2-RNAi* calli treated with BL (Supplementary Figure S4C). Therefore, these results further suggested that *MdJa2* gene played an important role in apple anthocyanin and proanthocyanidin synthesis.

### MdJa2 Binds to the *MdANS*, *MdMYB9* and *MdMYB12* Promoters

**Figure 4:**
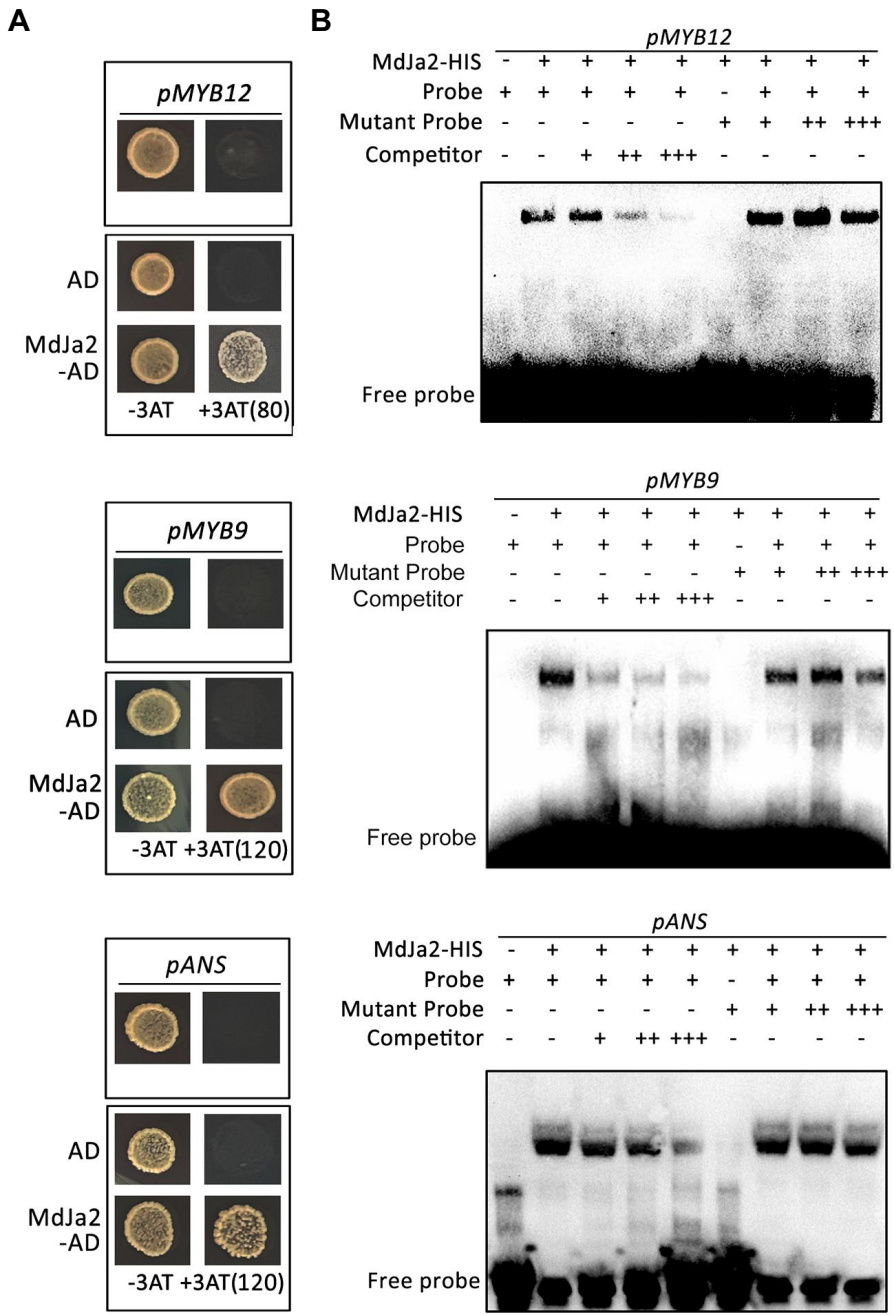
MdJa2 binds to the *MdANS*, *MdMYB9,* and *MdMYB12* promoters. **(A)** Yeast one-hybrid (Y1H) assays revealed the interactions between MdJa2 and *pANS*, *pMYB9*, and *pMYB12*. The pGADT7 + *ANS-pHIS2*, pGADT7 + *MYB9-pHIS2*, and pGADT7 + *MYB12-pHIS2* co-transformations served as the controls. The numbers in parentheses indicated the optimal 3-AT concentrations for suppressing the background histidine leakiness of the pHIS2 vectors. **(B)** Results of the EMSAs. The probe was a biotin-labeled fragment. The competitor probe was an unlabeled probe. The mutant probe contained two nucleotide mutations. Competitor and mutant probes at 50×, 100× and 200× molar excess were present (+) or absent (−) in each reaction. The bindings between MdJa2 and the *MdANS*, *MdMYB9* and *MdMYB12* promoters were weakened with increasing competition probes concentrations. When the probes were mutated two nucleotides in the binding sites, MdJa2 did not bind to the *MdANS*, *MdMYB9,* and *MdMYB12* promoters. However, when the mutant probes and the biotin-labeled probes were added together, their bindings between them were not affected.

The MADS-box transcription factors recognize the CArG motif in target gene promoters. In this study, yeast one-hybrid (Y1H) assays were conducted to assess the binding of MdJa2 to the promoters of anthocyanin and proanthocyanidin biosynthesis-associated genes. The *MdJa2* gene was inserted into the pGAD vector, whereas the *MdANS*, *MdMYB9*, and *MdMYB12* promoters were incorporated into the pHIS2 vectors. The promoters were screened using different concentrations of 3-AT (3-Amino-1,2,4-triazole; Supplementary Figure S5). The empty pGAD vector was used as a negative control. The assays indicated that MdJa2 could interact directly with the *MdANS*, *MdMYB9,* and *MdMYB12* promoters (Figure 4A). In the subsequent EMSA, the purified MdJa2-His fusion protein combined with specific fragments of the *MdANS*, *MdMYB9,* and *MdMYB12* promoters. The bindings between MdJa2 and the *MdANS*, *MdMYB9* and *MdMYB12* promoters were weakened with increasing competition probes concentrations. When the probes were mutated two nucleotides in the binding sites, MdJa2 did not bind to the *MdANS*, *MdMYB9* and *MdMYB12* promoters. However, when the mutant probes and the biotin-labeled probes were added together, their bindings between them were not affected (Figure 4B).

### Interaction Between MdJa2 and MdBZR1 Proteins

**Figure 5:**
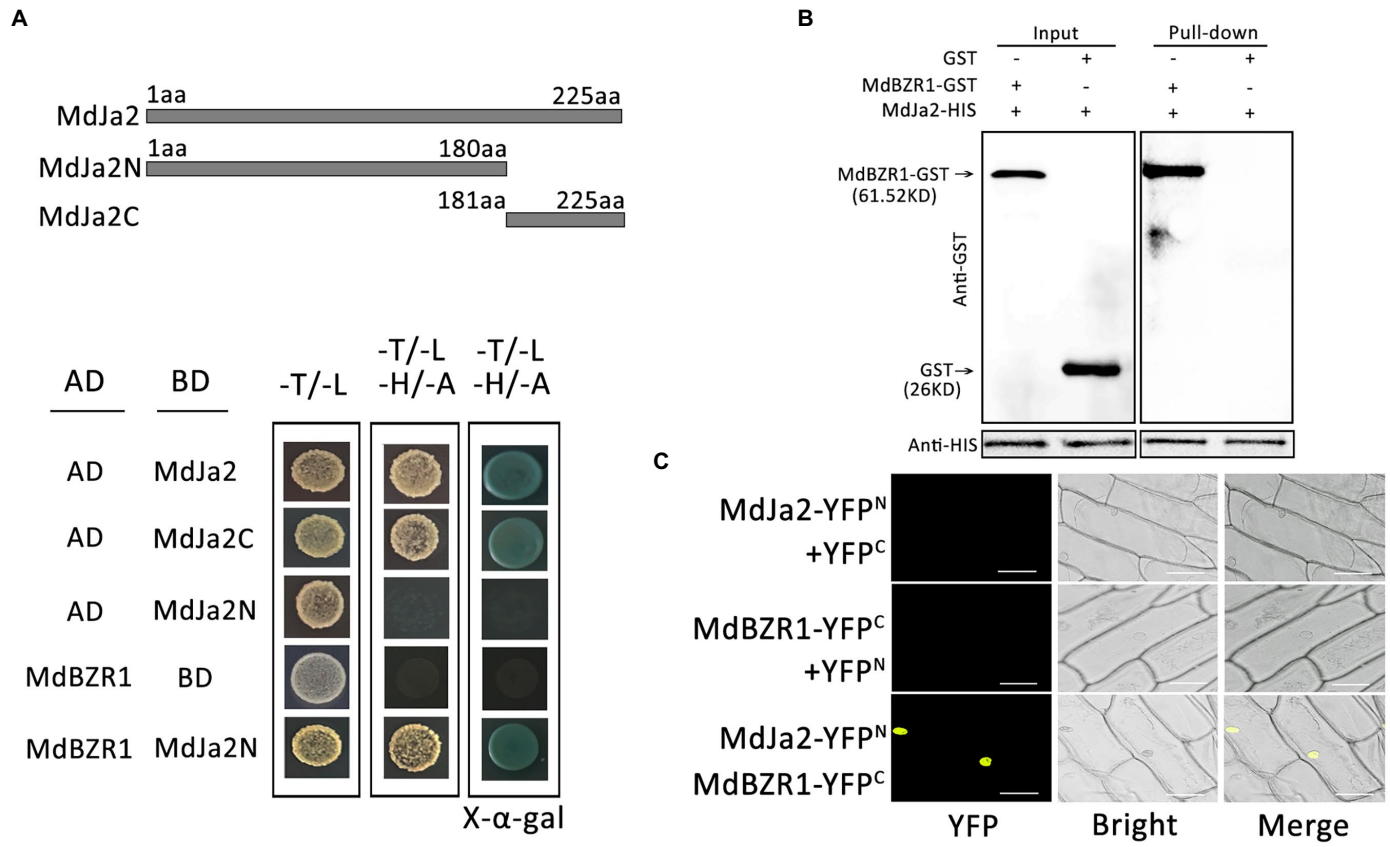
Interaction between MdJa2 and MdBZR1. **(A)** Schematic diagram of the MdJa2 protein structure. Yeast two-hybrid (Y2H) assays were used to analyze the interaction between MdJa2 and MdBZR1. The empty AD and BD vectors were used as controls. **(B)** Pull-down assays were performed by co-purifying the recombinant MdJa2-HIS fusion protein with MdBZR1-GST (column 1) and GST alone (column 2). A western blotting analysis involving an anti-GST antibody indicated that MdBZR1-GST was pulled down by MdJa2-HIS. **(C)** Confirmation of the interaction between MdJa2 and MdBZR1 *in vivo* according to a bimolecular fluorescence complementation assay. Scale bar = 50 μm.

The BZR1/BES1 transcription factors form a core element mediating BR signal transduction, which modulates the transcriptions of downstream target genes to induce multiple BR responses in plants. In this study, MdJa2 was confirmed to participate in the BR signaling pathway. To explore how it affects the BR regulatory pathway, we verified the interaction between MdJa2 and MdBZR1 through Y2H, pull-down, and Bimolecular Fluorescence Complementation (BiFC) assays. First, the MdJa2 sequences encoding the N-terminal (MdJa2N) and C-terminal (MdJa2C) domains were inserted into the pGBKT7 vector. The MdBZR1 cDNA sequence was inserted into the pGADT7 vector. Competent yeast cells were transformed with the recombinant plasmids and cultured on −Trp/−Leu medium and −Trp/−Leu/−Ade/−His medium in plates at 28°C. The yeast cells co-transformed with pGAD-MdBZR1 and pGBD-MdJa2N grew normally on the −Trp/−Leu/−Ade/−His medium (Figure 5A), reflecting the interaction between MdJa2 and MdBZR1. This interaction was confirmed in pull-down assays. Specifically, MdJa2-His pulled down the MdBZR1-GST fusion protein, but not GST alone (Figure 5B). Next, BiFC assays were performed to verify these results *in vivo*. Onion epidermal cells co-transformed with the MdJa2-YFP^N^ construct and the MdBZR1-YFP^C^ construct or the empty vector control (pSPYCE-35S) were examined. Strong yellow fluorescence was detected in the nucleus of onion cells containing MdJa2-YFP^N^ and MdBZR1-YFP^C^. Fluorescence was undetectable in the cells transformed with the empty vector (Figure 5C).

### *MdBZR1* Regulates Red-Fleshed Apple Anthocyanin and Proanthocyanidin Synthesis

**Figure 6:**
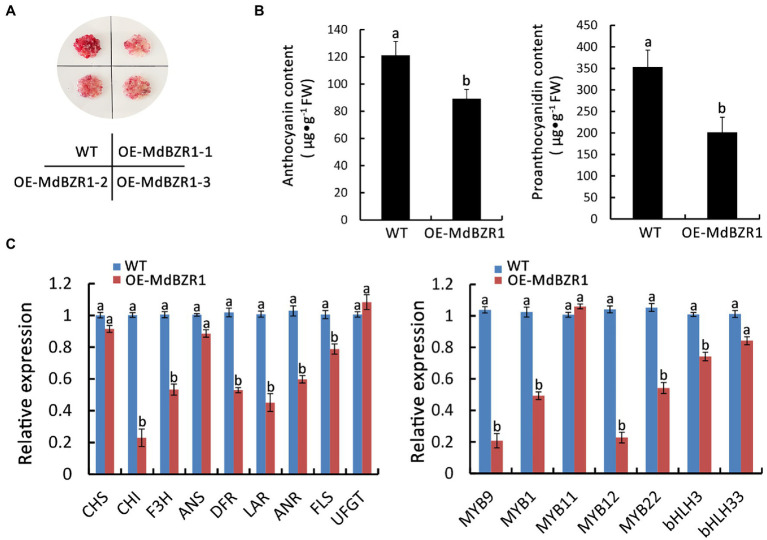
*MdBZR1* regulates the synthesis of anthocyanin and proanthocyanidin in red-fleshed apple calli. **(A)** Phenotypes of *OE-MdBZR1* red-fleshed apple calli. **(B)** Anthocyanin and proanthocyanidin contents in WT and transgenic lines. **(C)** Expressions of the genes related to flavonoid synthesis in *OE-MdBZR1* red-fleshed apple calli as determined by qRT-PCR. Statistical significance is indicated by different lowercase letters (*p < 0.05*).

To evaluate whether *MdBZR1* is involved in the synthesis of red-fleshed apple anthocyanin and proanthocyanidin, we overexpressed *MdBZR1* in WT red-fleshed apple calli. Three independent *MdBZR1-*overexpressing calli were selected for the gene expression analysis (Supplementary Figure S6). Compared with the WT calli, the coloration (Figure 6A) and the anthocyanin and proanthocyanidin contents (Figure 6B) decreased in the *MdBZR1*-overexpressing calli. To clarify the regulatory effect of *MdBZR1* on the transcriptions of genes in the flavonoid synthesis pathway, we analyzed the expressions of the flavonoid synthesis-related genes and transcription factor genes in *MdBZR1*-overexpressing red-fleshed apple calli. Compared with the control, the expression levels of the flavonoid synthesis structural genes (*MdDFR*, *MdLAR*, and *MdANR*) and transcription factor genes (*MdMYB9*, *MdMYB1* and *MdMYB12*) were down-regulated in the *MdBZR1*-overexpressing calli (Figure 6C). Accordingly, *MdBZR1* may function as an inhibitor in anthocyanin and proanthocyanidin synthesis pathways.

### The MdJa2–MdBZR1 Complex Affects Downstream Gene Transcriptions

**Figure 7:**
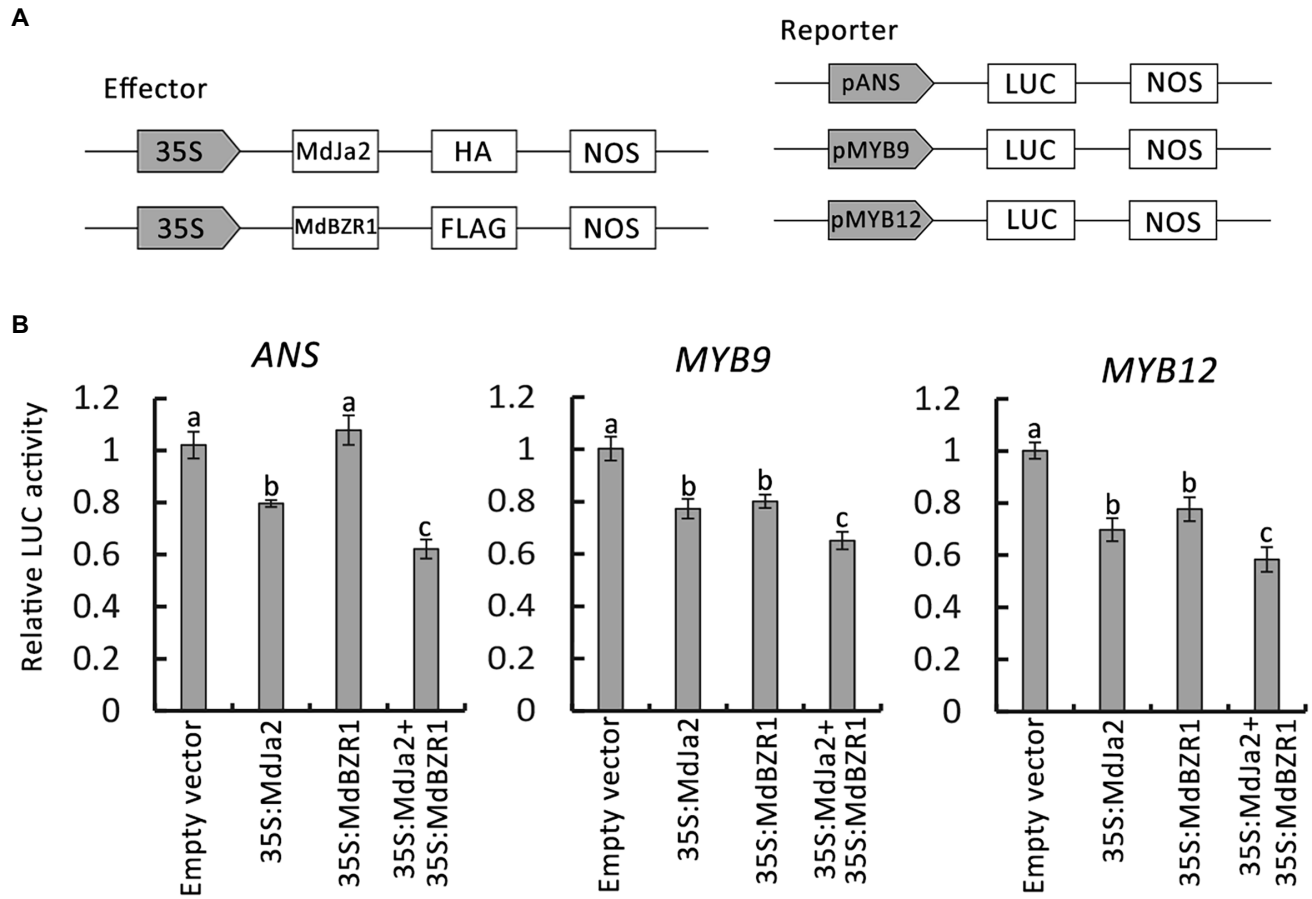
Effects of the MdBZR1–MdJa2 interaction on downstream genes. **(A)** Schematic diagrams of the LUC reporter vectors containing the *MdANS*, *MdMYB9* or *MdMYB12* promoters and the effector vectors containing *MdJa2* or *MdBZR1*. **(B)** Effects of the interaction between MdJa2 and MdBZR1 on the *MdANS*, *MdMYB9* and *MdMYB12* promoter’s activities as demonstrated by a luciferase reporter assay. The empty vector was used as a control. Statistical significance is indicated by different lowercase letters (*p < 0.05*).

The effects of the MdJa2–MdBZR1 complex on the downstream target genes promoter’s activities were determined by conducting LUC reporter assays. First, the *MdJa2* and *MdBZR1* sequences were inserted into the pHBT-HA and pHBT-FLAG vectors, respectively. The promoter sequences of the downstream target genes, including *MdANS*, *MdMYB9*, and *MdMYB12*, were inserted into the pFRK1-LUC vectors (Figure 7A). ‘Orin’ calli protoplasts were transformed with different combinations of the recombinant plasmids, with the empty vector used as a control. The results indicated that *MdJa2* could inhibit the transcriptions of the downstream target genes (*MdANS*, *MdMYB9* and *MdMYB12*). The inhibition was greater in the presence of both proteins than in the presence of only one of the proteins (Figure 7B). Therefore, the MdJa2–MdBZR1 complex could influence the transcriptions of key genes in the red-fleshed apple anthocyanin and proanthocyanidin synthesis pathways.

## Discussion

Because the molecular mechanism underlying BR signaling has been thoroughly characterized, BR has been widely used as a novel and efficient regulator to improve agricultural production. For example, the application of 5 μM BR effectively inhibits ethylene release in dates and enhances the resistance to *Penicillium* species by enhancing enzymatic activities (Zhu et al., 2010). In strawberries, BR is involved in the early fruit maturation process (Chai et al., 2013). However, there have been relatively few reports describing the regulatory effects of BR on plant flavonoid biosynthesis pathways. In this study, a 1 μM BL treatment decreased the anthocyanin and PA contents in red-fleshed apple seedlings and calli. Consistent with this finding, the expression levels of flavonoid pathway structural genes and important regulatory genes were down-regulated, these results were in line with those previously reported (Wang et al., 2021). In contrast, 24-Epibrassinolide could enhance 5-ALA-induced anthocyanin and flavonol accumulation in calli of “Fuji” apple flesh (Zheng et al., 2018). A BR spray application reportedly promotes fruit maturation as well as PA synthesis during the grape fruit-setting phase (Symons et al., 2006; Luan et al., 2013). Similarly, an exogenous BR treatment increases the secondary metabolite content and antioxidant capacity of *Arabidopsis* grown in darkness (Ghassemi-Golezani et al., 2020). Thus, the potential mechanisms underlying the regulatory effects of BR on flavonoid biosynthesis may vary among plant species and possibly due to the different concentrations of BR used, which may further enrich BR’s role in flavonoid biosynthesis.

Flavonoids play key roles throughout the plant life cycle and are vital for improving human health. There are three main subclasses of flavonoids, namely flavonols, anthocyanins, and PAs (Harborne and Williams, 2001). There is considerable interest in flavonoid synthesis and metabolism in fruits and vegetables. The MBW ternary complex is the main regulator of the associated pathway (Baudry et al., 2004; Quattrocchio et al., 2006; An et al., 2012), which is also mediated by many other transcription factors (e.g., MADS, NAC, HD-Zip, WRKY and HSF family members; Wu et al., 2014; Jiang et al., 2017; Liu et al., 2019; Sun et al., 2019; Wang et al., 2020). In this study, we cloned a MADS-box gene (*MdJa2*) and determined that it belonged to the STMADS11 subfamily and encoded a highly conserved MADS-box domain. We observed that the expression of *MdJa2* was regulated by BR signal. Additionally, the overexpression of *MdJa2* inhibited anthocyanin and proanthocyanidin synthesis in red-fleshed apple calli. These results indicated that *MdJa2* might be modulated by BR signaling to influence the synthesis of red-fleshed apple anthocyanin and PA.

**Figure 8:**
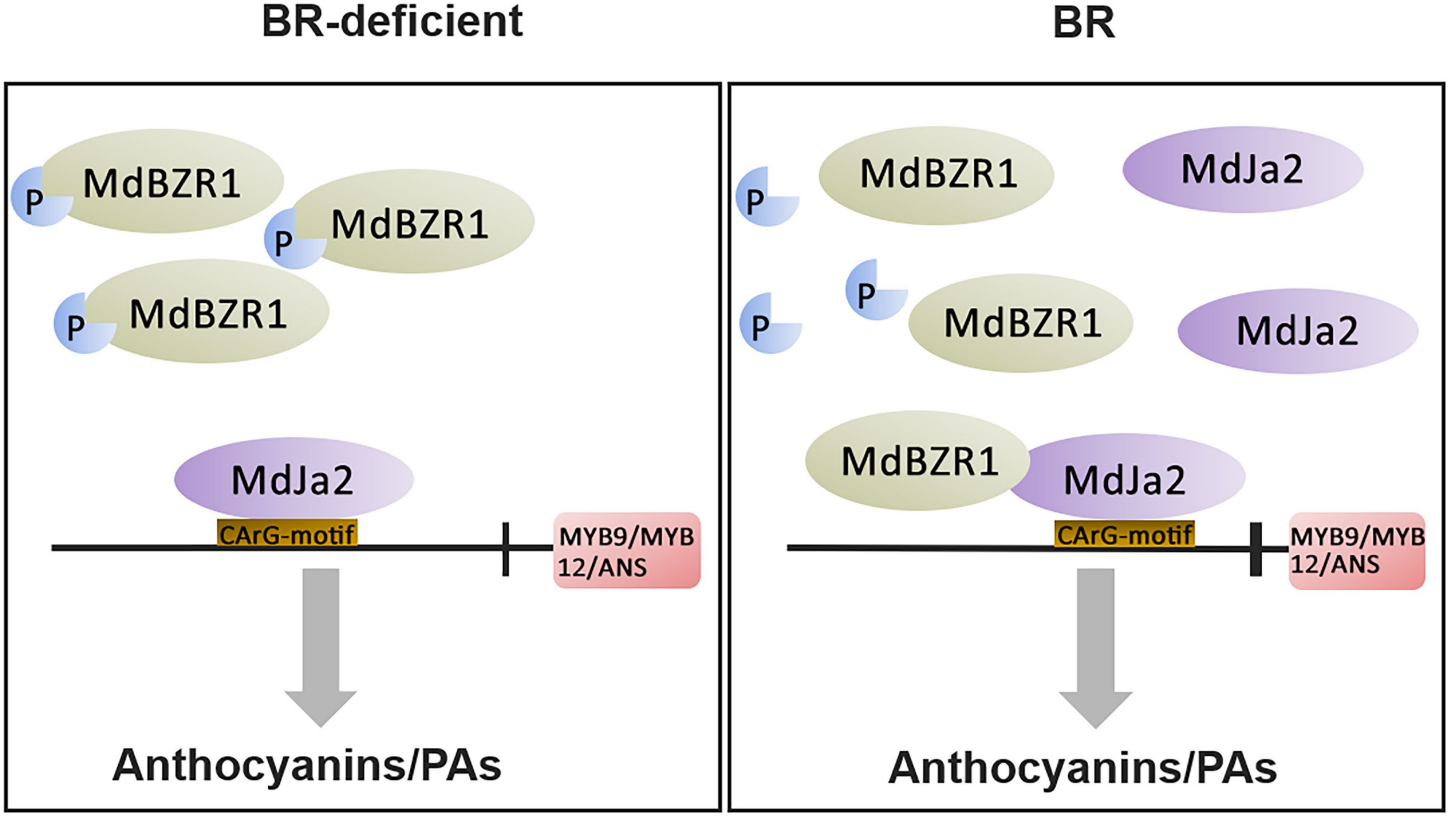
Proposed model for BR-regulated anthocyanin and proanthocyanidin biosynthesis mediated by the interaction between MdJa2 and MdBZR1. When plants are treated with BR, MdJa2 accumulates and most MdBZR1 proteins exist in a dephosphorylated active state, which considerably increases the possibility that MdBZR1 and MdJa2 will form a complex. This complex inhibits anthocyanin and PA synthesis by modulating the transcriptions of downstream target genes. P indicates phosphorylation modification, CArG-motif indicates binding element, vertical line indicates inhibition, and bold vertical line indicates enhanced inhibition.

As we all know, BZR transcription factors play important roles in regulating the BR response, and they can form complexes with other transcription factors. For example, BZR1 can interact with PIF4 to participate in the BR signaling pathway and cause yellowing of seedlings (Oh et al., 2012). In addition to BEH1, five BES1/BZR1 proteins can interact with MAX2 to activate strigolactone signaling (Wang et al., 2013). In apples, the MdBES1-MdMYB88 cascade transcription module regulates plant growth and tolerance to abiotic stress (Liu et al., 2021). In this study, we demonstrated that MdBZR1 interacted with MdJa2. Furthermore, MdJa2 bound to the *MdANS*, *MdMYB9,* and *MdMYB12* promoters. The formation of the MdBZR1–MdJa2 complex enhanced the ability of MdJa2 to bind to the promoters of *MdANS*, *MdMYB9,* and *MdMYB12*, which further down-regulated the expression of these genes. However, whether MdBZR1 can bind to the downstream structural genes of the anthocyanin and PA synthesis pathways will need to be determined in future investigations. The co-transformation of ‘Orin’ calli protoplasts with *MBZR1* and *MdJa2* clarified the effect of the MdBZR1–MdJa2 complex on anthocyanin and PA synthesis. When seedlings are grown in the absence of BR, the accumulation of MdJa2 decreases substantially and most MdBZR1 proteins exist in the phosphorylated inactive state, which greatly weakens the inhibitory effect of MdBZR1–MdJa2 on anthocyanin and PA synthesis. When seedlings are treated with BR, MdJa2 accumulates and most MdBZR1 proteins exist in a dephosphorylated active state, which considerably increases the possibility that MdBZR1 and MdJa2 will form a complex. This complex inhibits anthocyanin and PA synthesis by modulating the transcriptions of downstream target genes. Therefore, we developed a hypothetical model (Figure 8) explaining how *MdJa2* participates in BR signaling to regulate red-fleshed apple anthocyanin and PA biosynthesis.

## Conclusion

In conclusion, we used molecular and genetic methods to study the *MdJa2* regarding its involvement in the BR pathway and its regulatory effects on the synthesis of anthocyanin and PA in red-fleshed apple. These findings shed new light on the mechanisms controlling anthocyanin and PA accumulations in red-fleshed apple.

## Data Availability Statement

The original contributions presented in the study are included in the article/Supplementary Material, further inquiries can be directed to the corresponding author.

## Author Contributions

MS: preparation, creation and/or presentation of the published work, and specifically writing the initial draft (including substantive translation). SW, WL, MY, ZZ, and NW: preparation, creation and/or presentation of the published work by those from the original research group, specifically critical review, and commentary or revision including pre- or post-publication stages. XC: ideas, formulation or evolution of overarching research goals and aims, and acquisition of the financial support for the project leading to this publication. All authors contributed to the article and approved the submitted version.

## Funding

This study was supported by the Agricultural Variety Improvement Project of Shandong Province (2021LZGC024) and the National Natural Science Foundation of China (31730080).

## Conflict of Interest

The authors declare that the research was conducted in the absence of any commercial or financial relationships that could be construed as a potential conflict of interest.

## Publisher’s Note

All claims expressed in this article are solely those of the authors and do not necessarily represent those of their affiliated organizations, or those of the publisher, the editors and the reviewers. Any product that may be evaluated in this article, or claim that may be made by its manufacturer, is not guaranteed or endorsed by the publisher.

## Footnotes

^1^ http://www.novagen.com/

^2^ http://www.novagen.com/
