# Supplementary material for: MdJa2 Participates in the Brassinosteroid Signaling Pathway to Regulate the Synthesis of Anthocyanin and Proanthocyanidin in Red-Fleshed Apple
Source: Front Plant Sci. 2022 May 9;13:830349. doi: 10.3389/fpls.2022.830349 (PMC9125324; doi:10.3389/fpls.2022.830349)
Supplement: Supplementary file 4 [file Data_Sheet_1.docx]

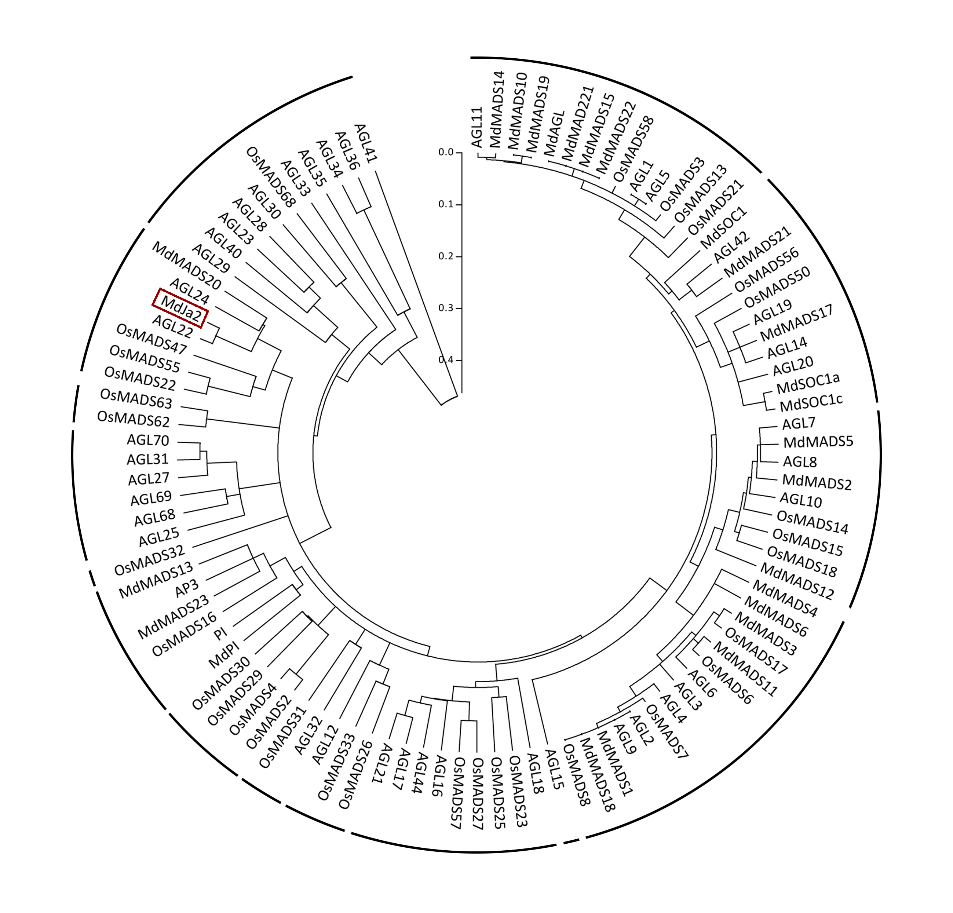


Fig. S1. Phylogenetic analysis of MdJa2. A phylogenetic tree comprising *Malus domestica*, *Arabidopsis thaliana*, and *Oryza sativa* L. MADS-box family proteins were constructed. All MADS-box protein sequences were obtained from the NCBI database (<https://www.ncbi.nlm.nih.gov/>).


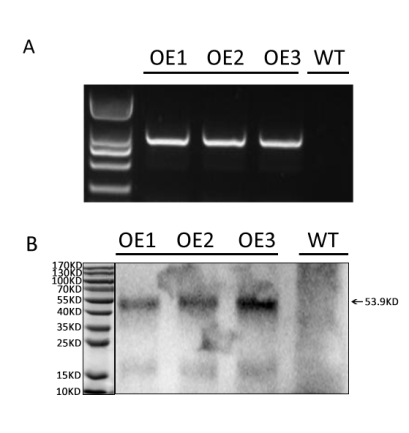


Fig. S2. Validation of *OE-MdJa2* transgenic red-fleshed apple calli by RT-PCR and western blotting analysis. The production of *OE-MdJa2* transgenic red-fleshed apple calli was confirmed by a RT-PCR amplification and a western blotting. Wild-type (WT) calli were used as controls.


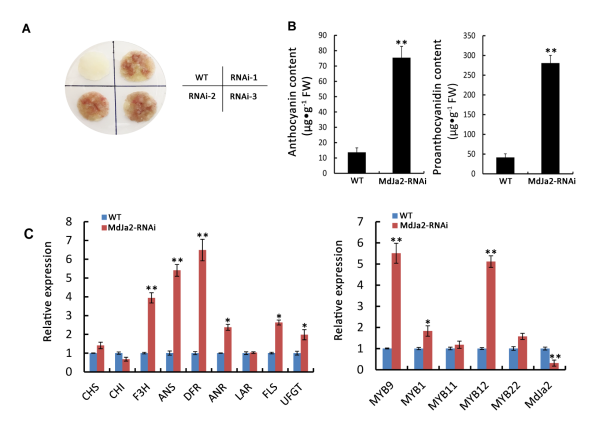


Fig. S3. The Silencing of *MdJa2* promotes anthocyanin and proanthocyanidin synthesis in ‘Orin’ calli. A. Phenotypes of *MdJa2-RNAi* ‘Orin’ calli under light conditions. B. Anthocyanin and proanthocyanidin contents in *MdJa2-RNAi* ‘Orin’ calli. C. Expressions of flavonoid synthesis-related genes in *MdJa2-RNAi* ‘Orin’ calli as determined by qRT-PCR. Statistical significance : * *P < 0.05*; ** *P < 0.01*.


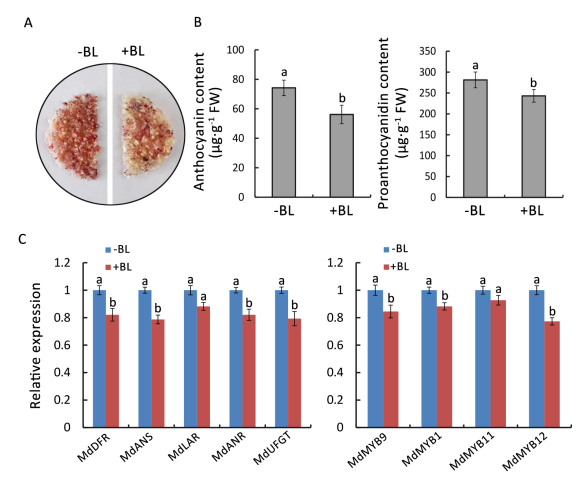


Fig. S4. Effect of BL-treatment on anthocyanin and proanthocyanidin synthesis in *MdJa2-RNAi* apple calli. A. Phenotypes of *MdJa2-RNAi* apple calli grown on MS medium with or without 1 μM BL. B. Anthocyanin and proanthocyanidin contents in *MdJa2-RNAi* apple calli with or without 1 μM BL. C. Expressions of the genes related to flavonoid synthesis in *MdJa2-RNAi* apple calli with or without 1 μM BL as determined by qRT-PCR. Statistical significance is indicated by different lowercase letters (*P < 0.05*).


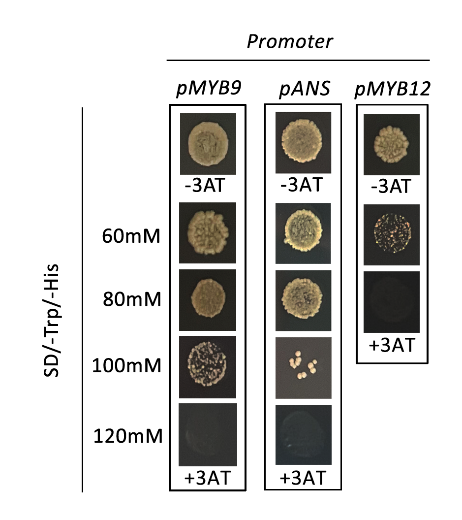


Fig. S5. Determination of the optimal 3-AT concentrations for the *ANS*, *MYB9*, and *MYB12* promoters. The numbers on the left indicate the different 3-AT concentrations for the interactions on the SD/−Trp/−His medium. The concentrations that prevented yeast strains from growing were suitable for suppressing the background histidine leakiness of the pHIS2 vectors.


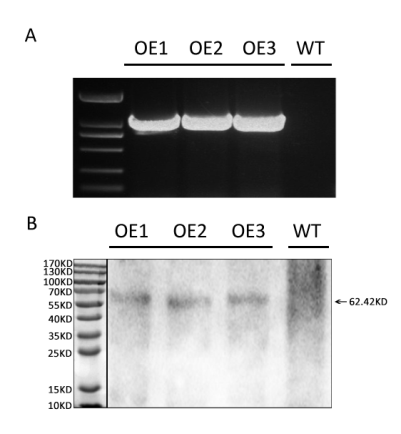


Fig. S6. Validation of *OE-MdBZR1* transgenic red-fleshed apple calli by RT-PCR and western blotting analyses. The production of *OE-MdBZR1* transgenic red-fleshed apple calli was confirmed by a RT-PCR amplification and a western blotting. Wild-type (WT) calli were used as controls.
